# Supplementary material for: Multi‐dataset identification of innovative feature genes and molecular mechanisms in keratoconus
Source: J Cell Mol Med. 2024 Sep 19;28(18):e70079. doi: 10.1111/jcmm.70079 (PMC11412914; doi:10.1111/jcmm.70079)
Supplement: Supplementary file 2 — Material S2. [file JCMM-28-e70079-s002.zip › 7.GSEA analysis/1.ARL11/KEGG_BASE_EXCISION_REPAIR.html]

Details for gene set KEGG\_BASE\_EXCISION\_REPAIR[GSEA]

|  || Dataset | ARL11.group.cls#h\_versus\_l |
| Phenotype | group.cls#h\_versus\_l |
| Upregulated in class | h |
| GeneSet | KEGG\_BASE\_EXCISION\_REPAIR |
| Enrichment Score (ES) | 0.3646268 |
| Normalized Enrichment Score (NES) | 1.146784 |
| Nominal p-value | 0.35490197 |
| FDR q-value | 1.0 |
| FWER p-Value | 0.995 |
Table: GSEA Results Summary

  

Fig 1: Enrichment plot: KEGG\_BASE\_EXCISION\_REPAIR      
 Profile of the Running ES Score & Positions of GeneSet Members on the Rank Ordered List

  

| SYMBOL | TITLE | RANK IN GENE LIST | RANK METRIC SCORE | RUNNING ES | CORE ENRICHMENT || 1 | NEIL3 | na | 19 | 0.920 | 0.3646 | Yes |
| 2 | POLE2 | na | 3717 | 0.211 | 0.3419 | No |
| 3 | OGG1 | na | 7376 | 0.104 | 0.2778 | No |
| 4 | POLE | na | 8165 | 0.089 | 0.2904 | No |
| 5 | UNG | na | 9899 | 0.061 | 0.2649 | No |
| 6 | HMGB1 | na | 11437 | 0.042 | 0.2374 | No |
| 7 | POLD1 | na | 11636 | 0.040 | 0.2475 | No |
| 8 | PCNA | na | 11742 | 0.039 | 0.2599 | No |
| 9 | APEX1 | na | 12980 | 0.027 | 0.2351 | No |
| 10 | POLL | na | 13157 | 0.026 | 0.2401 | No |
| 11 | PARP4 | na | 13251 | 0.025 | 0.2473 | No |
| 12 | TDG | na | 13746 | 0.021 | 0.2414 | No |
| 13 | NEIL1 | na | 13848 | 0.020 | 0.2464 | No |
| 14 | POLD4 | na | 16051 | 0.003 | 0.1843 | No |
| 15 | LIG3 | na | 16228 | 0.002 | 0.1801 | No |
| 16 | LIG1 | na | 16850 | -0.002 | 0.1631 | No |
| 17 | APEX2 | na | 18114 | -0.011 | 0.1311 | No |
| 18 | POLD3 | na | 18840 | -0.016 | 0.1165 | No |
| 19 | MUTYH | na | 19422 | -0.020 | 0.1077 | No |
| 20 | SMUG1 | na | 19430 | -0.020 | 0.1154 | No |
| 21 | PARP2 | na | 19614 | -0.021 | 0.1185 | No |
| 22 | MBD4 | na | 20006 | -0.024 | 0.1167 | No |
| 23 | POLE3 | na | 20917 | -0.031 | 0.1026 | No |
| 24 | XRCC1 | na | 21628 | -0.036 | 0.0965 | No |
| 25 | MPG | na | 22394 | -0.044 | 0.0919 | No |
| 26 | POLE4 | na | 22854 | -0.049 | 0.0979 | No |
| 27 | PARP3 | na | 22860 | -0.049 | 0.1171 | No |
| 28 | NTHL1 | na | 23911 | -0.061 | 0.1110 | No |
| 29 | POLD2 | na | 24003 | -0.062 | 0.1331 | No |
| 30 | NEIL2 | na | 25538 | -0.083 | 0.1220 | No |
| 31 | PARP1 | na | 25603 | -0.084 | 0.1536 | No |
| 32 | POLB | na | 27681 | -0.123 | 0.1426 | No |
| 33 | FEN1 | na | 28815 | -0.152 | 0.1704 | No |
Table: GSEA details [plain text format]

  

Fig 2: KEGG\_BASE\_EXCISION\_REPAIR      
 Blue-Pink O' Gram in the Space of the Analyzed GeneSet

  

Fig 3: KEGG\_BASE\_EXCISION\_REPAIR: Random ES distribution      
 Gene set null distribution of ES for **KEGG\_BASE\_EXCISION\_REPAIR**
